# Factors on educational outcome for obesity prevention in female adolesecents in Korea

**DOI:** 10.1186/1687-9856-2013-S1-P103

**Published:** 2013-10-03

**Authors:** Hyejung Shin, Seolhyang Baek

**Affiliations:** 1 Department of Pediatrics, National medical center, South Korea Department of Nursing, College of medicine, Dongguk University, South Korea

## Aims

High demands for academic achievement seem to lead adolescents to study more but exercise less, which eventually increases the prevalence of youth obesity whose health status might be poorer than a few decades ago. As a way of cutting off obesity prevalence in high school students in Korea, this study aims to measure the association between health status, daily behaviors and educational outcome.

## Methods

276 female high school students in Seoul were enrolled to attend the obesity education in their physical classroom. Mean age was 17.2±0.5 years old. We obtained weight, height, waist circumference. The obese, overweight was defined as body mass index(BMI) more than 95th, 85th~94th percentile respectively for age and sex. All respondents were asked to answer a structured checklist of family history, past history, review of symptoms, and health-related behavior before the education class, and the other questionnaire was given twice to measure the obesity-related behaviors at the same time and one month later.

## Results

The number of adolescents with obesity was 33(12.0%). 20(7.24%) subjects belonged to overweight group. 222(80.4%)students showed improvement on obesity-related behaviors after education. The number of physical health problems for recent 1 year was significantly correlated with the number of family history(r=0.20, p =0.001) and the number of current physical symptoms for recent 1 month(r=0.27, p<.001). Daily unhealthy behavior for recent 1 month was not only correlated with the number of current physical symptoms for recent 1 month(r=0.28, p<0.001) but also mental health symptoms for recent 1 month(r=0.36, p<=0.001). In the multivariate linear regression model, daily healthy behavior for recent 1 month only showed significant impact on educational outcome with odds ratio 0.81 (95% C.I. : 0.661-0.992).

## Conclusion

Educational program for obesity prevention improved daily healthy behaviors on survey after one month. Daily healthy behavior for recent 1 month was associated with educational outcome.

